# Impact of the MEDEA exposure-reduction strategies on indoor air quality during desert dust storms

**DOI:** 10.1093/eurpub/ckac130.066

**Published:** 2022-10-25

**Authors:** S Achilleos, A Michanikou, P Kouis, SI Papatheodorou, A Panayiotou, P Kinni, N Middleton, P Koutrakis, PK Yiallouros

**Affiliations:** 1 Department of Primary Care & Population Healt, University of Nicosia Medical School, Nicosia, Cyprus; 2 Cyprus International Institute for Environmental and Public Health, Cyprus University of Technology, Limassol, Cyprus; 3 Respiratory Physiology Laboratory, University of Cyprus Medical School, Nicosia, Cyprus; 4 Department of Epidemiology, School of Public Health, Harvard University, Boston, MA, USA; 5 Department of Nursing, Cyprus University of Technology, Limassol, Cyprus; 6 Department of Environmental Health, School of Public Health, Harvard University, Boston, MA, USA

## Abstract

**Background:**

Desert dust storms (DDS) impact the Mediterranean basin heavily, particularly in the context of climate change, reduced precipitation and increasing desertification. There is a pressing need to develop policies protecting EU citizens’ health from DDS. While Public Health authorities in affected regions commonly issue warnings, the effectiveness of recommendations to reduce exposure has not been documented.

**Methods:**

This work is part of the wider “MEDEA” intervention studies, co-funded by LIFE 2016 Programme. Among other outcomes, the studies examined the effectiveness of an indoor exposure-reduction intervention (i.e., decrease home ventilation and use of air cleaners) across homes of asthmatic schoolchildren and individuals with atrial fibrillation in Cyprus. Participants were randomized to either a control or indoor intervention group. The assessment took place in a sample of participants’ homes, during 2019 and 2021, with the collection of indoor and outdoor PM10 and PM2.5 samples, which were analyzed for mass and elemental concentrations.

**Results:**

Indoor PM2.5 and PM10 mass and elements concentrations were significantly lower in the indoor intervention group compared to the control group, both during days with no dust (e.g., 55% and 48% reduction for PM2.5 and PM10, respectively) and days with desert dust (PM2.5: 47% and PM10: 40% reduction). In addition, the infiltration of PM2.5 and PM10 particles from the outdoor to the indoor air was significantly lower in the intervention vs. the control group (PM2.5: -55%, 95% CI: -42%, -65%; PM10: -41%, 95% CI: -61%, -12%).

**Conclusions:**

The study assessed a realistic exposure-reduction strategy and provided first-time evidence that closing doors and windows along with air cleaners can reduce indoor exposure to DDS particles. This evidence can further inform decision-making and strategic planning for population-level mitigation of DDS health effects in Mediterranean Europe.

**Key messages:**

- Decreasing home ventilation and use of air cleaners during DDS can reduce indoor particle mass concentrations by up to 50%, and infiltration of particles from outdoor environments by up to 60%.
- Demonstration of the effectiveness of a sustainable exposure-reduction intervention can contribute towards the adaptation of the population to the effects of climate change.

